# Downregulation of Endothelin Receptor B Contributes to Defective B Cell Lymphopoiesis in Trisomy 21 Pluripotent Stem Cells

**DOI:** 10.1038/s41598-018-26123-y

**Published:** 2018-05-22

**Authors:** Glenn A. MacLean, Jennifer McEldoon, Jialiang Huang, Jeremy Allred, Matthew C. Canver, Stuart. H. Orkin

**Affiliations:** 1 Division of Hematology/Oncology, Boston Children’s Hospital and Dana Farber Cancer Institute, Harvard Medical School Boston, MA 02115 Boston, USA; 2 Harvard Stem Cell Institute, Harvard Medical School Boston, MA 02115 Boston, USA; 3 Howard Hughes Medical Institute, Harvard Medical School Boston, MA 02115 Boston, USA

## Abstract

Individuals with Trisomy 21 (T21) exhibit numerous hematological abnormalities, including reductions in numbers of circulating B and T lymphocytes. To elucidate molecular mechanisms underlying these phenotypes, we differentiated human isogenic disomic and trisomic pluripotent cells, and observed that trisomic cells showed defects in B cell, but not T cell differentiation. Global gene expression of differentiated, trisomic B cells revealed reduced expression of genes encoding endothelin signaling components, namely the *Endothelin Receptor B (EDNRB)*, and its ligand *Endothelin1 (EDN1)*. Depletion of *EDNRB* mRNA in cord blood-derived CD34^+^ cells led to defective B cell differentiation, supporting a hypothesis that low *EDNRB* expression in T21 contributes to intrinsic lymphoid defects. Further evidence for the role of the *EDNRB* pathway in B cell differentiation was obtained through CRISPR/Cas9 gene targeting in disomic and trisomic iPS cells. Knockout of *EDNRB* in both cell backgrounds reduced the capacity for B cell differentiation. Collectively, this work identifies downregulation of *EDNRB* as a causative factor for impaired B lymphocyte generation in trisomic cells, which may contribute to defects in immune function associated with T21. Furthermore, a novel role for endothelin signaling in regulation of B cell development has been identified.

## Introduction

Trisomy 21 (T21), the most common viable chromosomal abnormality, has an incidence of ~1 in 700 in the United States^1^. Often caused by maternal chromosomal non-disjunction, T21 is characterized by phenotypes affecting numerous tissues, including craniofacial abnormalities, shortened extremities, cognitive impairment, heart defects and increased incidence of Hirschsprung Disease^2,3^. T21 is also associated with hematologic phenotypes, including an increase in fetal liver hematopoietic progenitor cells, and increased incidence of acute megakaryoblastic leukemia (AMKL) and B-cell acute lymphoblastic leukemia (B-ALL)^4–7^. T21 individuals also exhibit reduced numbers of circulating B and T lymphocytes^8–11^, which likely contributes to increased frequency of respiratory infections, altered vaccine responses and autoimmune disorders, which in aggregate affect morbidity and quality of life^12–14^.

The hematologic phenotypes of T21 are of particular interest as T21 may impact hematopoiesis at multiple steps in development. Though investigators have employed several mouse models for T21, such models fail to accurately recapitulate the diverse hematologic phenotypes observed in humans, and thus may be of limited utility in uncovering molecular mechanisms underlying T21 phenotypes^15^. These limitations provide a strong rationale for the use of differentiated human pluripotent cells to model aspects of T21, including perturbed hematopoiesis. Differentiated human ES and iPS cells have been used to model hematologic malignancies with varied success^16,17^. A significant caveat with *in vitro* disease modeling studies using differentiated pluripotent cells is inherent cell line variability in differentiation capacity. The use of isogenic iPS cells mitigates this problem, and is critical for exploring genetic effects in the absence of confounding genetic backgrounds.

We have isolated and characterized isogenic iPS cells that are either disomic or trisomic for chromosome 21. Isogenic subclones were originally isolated from a parental trisomic iPS line that spontaneously lost a copy of chromosome 21 in some cells^18^. These cells provide a unique tool for *in vitro* differentiation experiments that allow for determination of the effect of T21 in isolation^18^. We, and others, have demonstrated that differentiated trisomic pluripotent cells exhibit an increase in hematopoietic progenitor cells capable of multilineage colony forming potential^18–20^. Prior studies focused on megakaryocyte, erythroid and myeloid lineages, and recapitulated T21 phenotypes reported *in vivo*^6,5^. As the numbers of circulating B and T lymphocytes are reduced in T21 individuals, we have now extended analysis to *in vitro* lymphoid differentiation in an effort to uncover potential intrinsic deficits.

Trisomic clones exhibited a striking reduction in their capacity for B cell differentiation, yet T cell generation appeared unaffected. Transcriptomic analysis of differentiated B cells identified both a ligand (Endothelin 1) and receptor (Endothelin Receptor B) involved in Endothelin signaling as reduced in expression in trisomic cells. shRNA knockdown and CRISPR/Cas9 gene editing experiments further support the hypothesis that reduced *EDNRB* expression contributes to impaired B cell differentiation. Collectively, our findings identify EDNRB as a factor underlying the defect in B cell development seen in T21, and demonstrate that endothelin signaling is critical for proper B cell lymphopoiesis.

## Results

### Trisomy 21 subclones exhibit reduced capacity for B-cell differentiation

**Figure 1:**
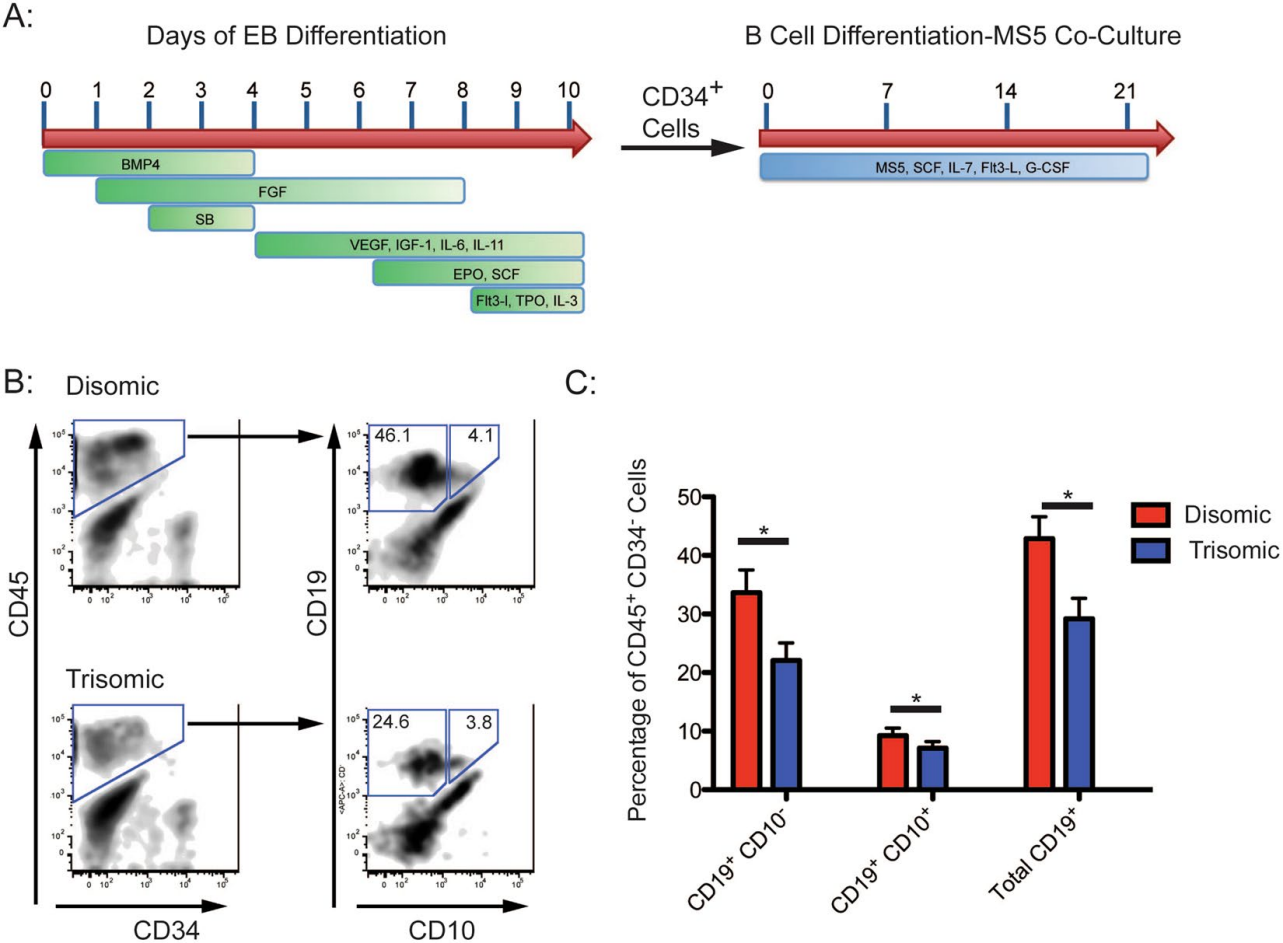
Trisomy 21 Pluripotent Cells Exhibit a Reduced Capacity for B Cell Differentiation. (**A**) Schematic of culture conditions for B Cell differentiation of human pluripotent cells. (**B**) Representative FACS plots of differentiated disomic and trisomic isogenic iPS cells. Plots show the distribution of CD19^+^CD10^+^ cells within gated CD45^+^CD34^−^ cells. (**C**) Quantification of FACS analysis, shown as percentage of CD19^+^ and CD10^+^ cells within CD45^+^CD34^−^ populations. Trisomic clones (N = 22) generated fewer CD19^+^ and CD19^+^CD10^+^ cells than disomic (N = 22) counterparts. (*p-value ≤ 0.05).

We employed previously characterized isogenic disomic and trisomic iPS cells^18^, to assess B lymphoid cell development. Undifferentiated cells were induced to hematopoietic differentiation via embryoid bodies^21^, and after 9–10 days of culture, hematopoietic progenitor cells expressing CD34^+^ were isolated and plated on MS5 feeder cells with cytokines permissive for B cell differentiation (Fig. 1A). After 3 weeks of co-culture with MS5 cells, cells were isolated and analyzed by FACS for expression of markers of B cell development. Disomic and trisomic iPS cells yielded similar numbers of CD45^+^CD34^−^ progenitors. In contrast, generation of developing B cells, encompassed by CD19^+^CD10^+^ and CD19^+^CD10^−^ populations, was reduced from trisomic iPS cells. Within this population, trisomic clones generated 22.0 ± 3.0% CD19^+^CD10^−^ and 7.1 ± 1.1% CD19^+^CD10^+^ cells, whereas disomic counterparts yielded 33.6 ± 3.9% CD19^+^CD10^−^ and 9.2 ± 1.3% CD19^+^CD10^+^ (Fig. 1B,C). The total percentage of CD19^+^ cells generated was 29.2 ± 3.5% in trisomic clones, and 42.9 ± 3.7% in disomic clones (Fig. 1C). These results are reminiscent of reduced numbers of B lymphoid cells described in T21 individuals, as well as in T21 fetal liver^8,22^.

### T lymphoid cell generation is unaffected from trisomy 21 iPS cells

**Figure 2:**
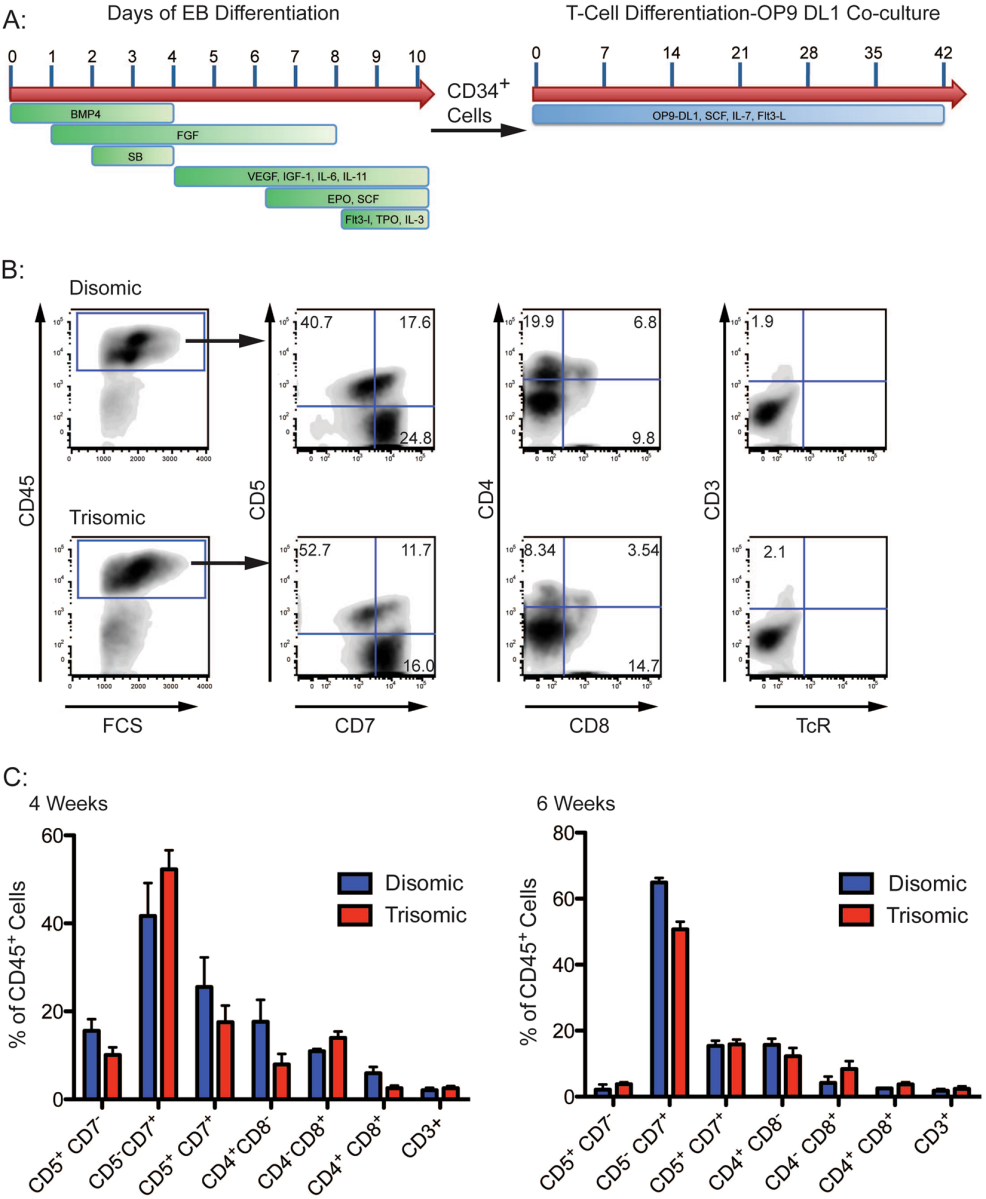
Disomic and Trisomic Isogenic iPS cells Show Comparable Efficiency in T Cell Differentiation. (**A**) Differentiation conditions for generation of T cells from isogenic iPS cells. (**B**) FACS analysis showing distribution of CD5/CD7, CD4/CD8 and CD3/TcRα/β cells within CD45^+^ populations. (**C**) Quantification of FACS analysis for T cell progenitor populations, no significant differences were observed between disomic and trisomic clones for any population analyzed after either 4 or 6 weeks of differentiation.

T-cell potential of isogenic disomic and trisomic iPS cells was evaluated by placing *in vitro* generated CD34^+^ cells into T cell differentiation assays (Fig. 2A)^21^. Following 4 or 6 weeks of co-culture, cells were analyzed by FACS to determine the extent of T-cell differentiation (Fig. 2B,C). Generation of early T-cell progenitor populations, defined by expression of CD5 and CD7, was similar between disomic and trisomic iPS cells at either 4 or 6 weeks of differentiation (Fig. 2B,C). Similarly, CD4 and CD8 single and double positive cells were detected at comparable levels (Fig. 2B,C). A small population of CD45^+^CD3^+^ cells was detected with no appreciable differences between disomic and trisomic subclones (Fig. 2B,C), thus indicating that T21 does not appear to affect T-cell development in this culture system. Taken together, despite reports of T cell defects in T21 individuals, *in vitro* T cell development appeared ostensibly unaffected^9,10^.

### Differentiated trisomic CD19^+^ B cells exhibit reduced expression of endothelin signaling genes

**Figure 3:**
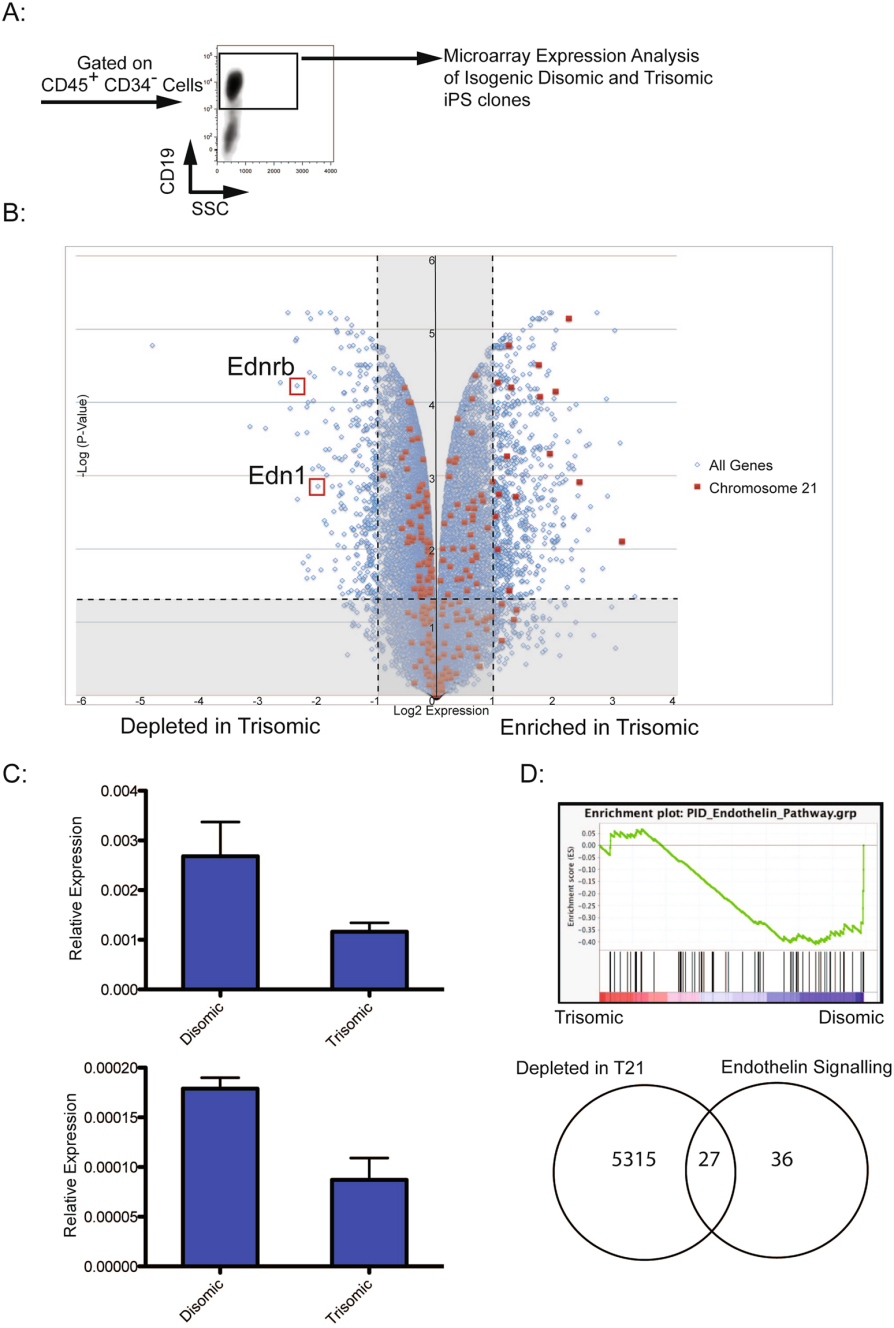
Global Gene Expression Analysis of Differentiated B Cells. (**A**) Gating profile of CD45^+^CD34^−^CD19^+^ cells isolated for expression analysis. (**B**) Volcano plot showing distribution of expressed genes in differentiated trisomic cells relative to disomic cells. Log_2_ (fold-change) is displayed on the X-axis, and Log (Adjusted P-Value) is along the Y-axis. Red squares represent genes on chromosome 21, and blue squares represent all other genes. (**C**) qPCR validation of *EDNRB* and *EDN1* expression in differentiated disomic and trisomic cells (N = 3 for each). Both genes are downregulated in trisomic cells, validating microarray results. (**D**) Gene Set Enrichment Analysis with a Endothelin Pathway gene set showing enrichment in differentiated disomic cells. A Venn diagram was constructed using the same Endothelin Pathway gene set, and genes depleted in trisomic clones by Log(fold change) <0.2, to indicate that 43% of the genes in the set are also depleted in differentiated trisomic clones (P-value < 0.0001).

In an effort to uncover mechanisms responsible for reduced B cell development from T21 iPS cells, global gene expression analysis was performed (Fig. 3A) on *in vitro* differentiated CD19^+^ cells. Comparison of transcriptomic profiles of differentiated disomic and trisomic subclones revealed 556 up-regulated genes and 286 genes downregulated genes in trisomic cells (fold change ≥2, p < 0.05) (Fig. 3B). In trisomic cells, a general enrichment in expression of chromosome 21 genes was observed, comparable to reports from other T21 gene expression studies^19,23^. Among chromosome 21 genes expressed in trisomic clones, we did not identify any candidates previously suspected as regulators of B cell lymphopoiesis. However, upon assessment of all transcripts, we observed that expression of both an endothelin receptor (*EDNRB*) and its ligand (*EDN1*) were reduced in trisomic clones (Fig. 3B, Supplementary Tables 1 and 2). Quantitative RT-qPCR confirmed microarray results (Fig. 3C). *EDNRB* encodes a G-protein coupled receptor that binds EDN1^24^. Gene Set Enrichment Analysis (GSEA) revealed that both G-protein_coupled_receptor_activity and Endothelin_Pathway gene sets were enriched in disomic cells (Figs 3D and S1). Overall, 43% of genes in the Endothelin Pathway gene set were downregulated in trisomic clones (Fisher exact test, P-value < 0.0001). A comparable overlap between the Endothelin Pathway gene set and genes enriched in trisomic clones was not observed (Supplementary Fig. S1b). A similar trend was observed with the G protein coupled receptor activity gene set (Supplementary Fig. S1a,b). Of potential relevance to our findings, mice deficient for *EDNRB* exhibit defects in B cell development^25,26^. Therefore, we hypothesized that reduced endothelin signaling may account in part for the observed abnormalities in B cell differentiation due to T21.

### Knockdown of *EDNRB* impairs B cell differentiation

**Figure 4:**
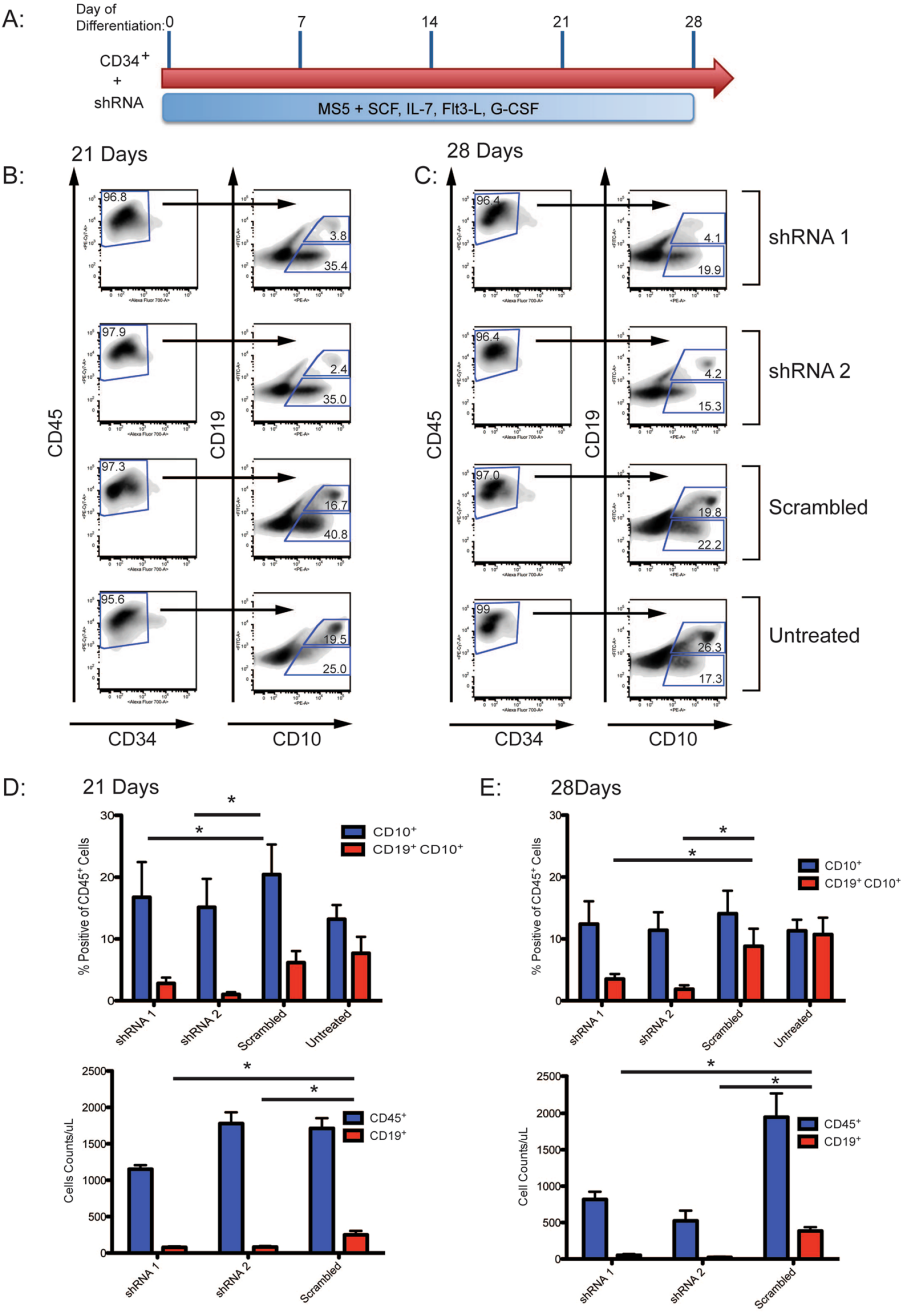
shRNA Knockdown of *EDNRB* in CB CD34^+^ cells Reduces Efficiency and Absolute Output of B Cell Differentiation. (**A**) Differentiation conditions for transduced CB CD34^+^ cells. (**B**,**C**) Representative FACS analysis of CB CD34^+^ cells after 21 or 28 days of differentiation. The first column of panels depicts CD45^+^ and CD34^+^ staining, and the second column shows CD19^+^ and CD10^+^ staining within the CD45^+^CD34^−^ population. Each row shows cells transduced with specific *EDNRB* shRNA, a scrambled control, or untransduced cells. (**D**,**E**) Quantification of FACS analysis showing percentage of CD10^+^ and CD19^+^CD10^+^ within the CD45^+^CD34^−^ population for various experimental groups after 21 and 28 days of differentiation. Also represented are the cell counts for generated CD45^+^ and CD19^+^ cells as determined using CountBright beads. (*p-value ≤ 0.05).

We focused attention on the role of *EDNRB* expression on B cell differentiation, as we reasoned that reduced expression of a receptor (*EDNRB*) rather than the ligand (*EDN1*) would be more limiting in establishing a phenotype. We tested 5 shRNAs directed to *EDNRB* for knockdown efficiency in HEK 293 T cells. Two shRNAs, NM_000115.2-1527s21c1 (shRNA1) and NM_000115.2-1323s21c1 (shRNA2), reduced *EDNRB* expression by 58% and 82%, respectively (Supplementary Fig. S2), and were employed in subsequent differentiation experiments. Normal disomic umbilical cord blood (CB) CD34^+^ cells were co-cultured with MS5 cells for 21–28 days (Fig. 4A). Under these conditions, the majority of cells differentiate to a CD45^+^CD34^−^ immunophenotype. As cells undergo B cell differentiation, they express CD10 followed by CD19^27^. To assess the impact of *EDNRB* knockdown on B cell differentiation, CB CD34^+^ cells were transduced with lentivirus encoding either a specific *EDNRB* shRNA or a scrambled shRNA control. At 21 days of co-culture, cells transduced with the two *EDNRB*-specific shRNAs generated 16.74 ± 5.70% and 15.11 ± 4.62% of CD10^+^ CD19^−^ cells, and 2.79 ± 0.95% and 1.01 ± 0.38% of CD10^+^CD19^+^ cells, respectively. In contrast, cells treated with scrambled shRNA control exhibit comparable levels of CD19^+^CD10^−^ cells (20.42 ± 4.87%), but a greater number of CD19^+^CD10^+^ cells (6.15 ± 1.90%) (Fig. 4D). After 28 days of co-culture, cells treated with *EDNRB* shRNA also showed significant reductions in production of CD10^+^CD19^+^ positive cells in comparison to control cells transduced with scrambled shRNA (3.51 ± 0.82% and 1.86 ± 0.65% compared to 8.80 ± 2.84%) (Fig. 4E). The total number of cells generated by *in vitro* differentiation was also quantitated. At 21 days of co-culture, samples treated with specific *EDNRB* shRNA generated fewer CD19^+^ cells than controls exposed to scrambled shRNA (76.99 ± 16.16 and 82.86 ± 13.05 cells/μL compared to 248.42 ± 56.2 cells/μL) (Fig. 4D). Similarly, after 28 days of co-culture, shRNA transduced cells generated fewer CD45^+^ cells (818.05 ± 105.44 and 523.53 ± 140.06 cells/μL) and CD19^+^ cells (50.71 ± 16.77 and 22.51 ± 9.19 cells/μL) than control samples (1947.02 ± 327.25 CD45^+^ cells/μL and 383.67 ± 53.45 CD19^+^ cells/μL) (Fig. 4E). These results demonstrate that depletion of *EDNRB* impairs B cell generation, and are consistent with the hypothesis that reduced expression contributes to impaired B cell production in T21.

### CRISPR/Cas9 targeting of *EDNRB* in isogenic iPS cells validates a role for *EDNRB* in B cell development

**Figure 5:**
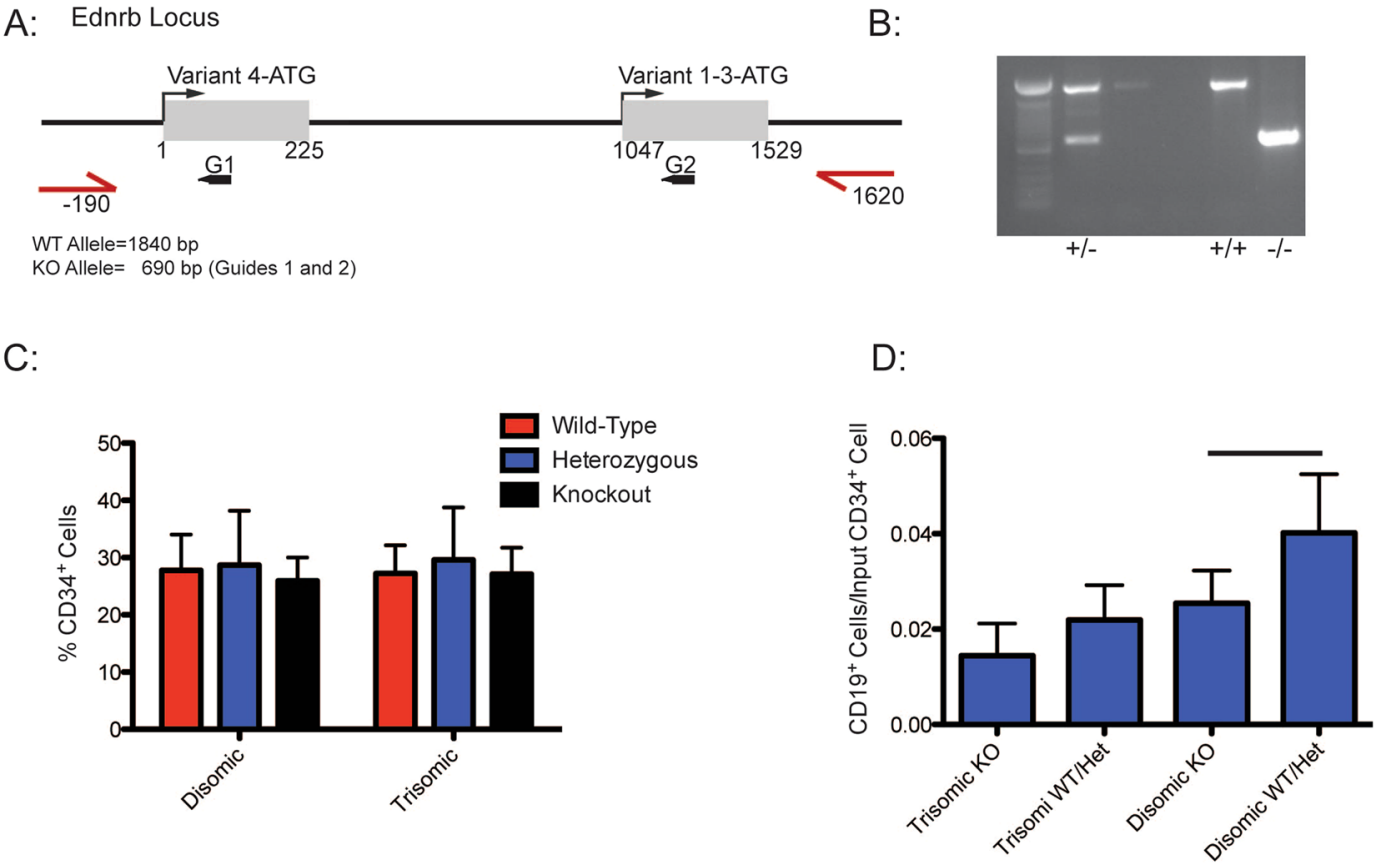
CRISPR/Cas9 Targeting of *EDNRB* in isogenic iPS cells. (**A**) Structure of the *EDNRB* locus showing localization of two guides that were used to ablate all 4 isoforms. (**B**) Genotyping of isogenic clones after CRISPR/Cas9 editing showing *EDNRB*^+/+^, *EDNRB*^+/−^ and *EDNRB*^−/−^ clones. (**C**) FACS analysis for CD34^+^ generated from Cas9 edited disomic and trisomic isogenic cells. (**D**) Quantification of B cell production in differentiated cells, presented as number of B cells generated per input CD34^+^ cell (p-value ≤ 0.15, between disomic KO and disomic WT/Het). G1 and G2 refer to two guides that were used for *EDNRB* editing.

In order to validate observations from shRNA knockdown of *EDNRB* in CB CD34^+^ cell differentiation, we ablated *EDNRB* expression by CRISPR/Cas9 in isogenic iPS cells. The *EDNRB* gene encodes 4 transcripts, with 2 alternative start codons. Hence, a dual sgRNA strategy to engender a genomic deletion was utilized to eliminate all isoforms (Fig. 5A). Undifferentiated disomic and trisomic isogenic iPS cells were electroporated with plasmids encoding Cas9, sgRNAs 1 and 2, and a GFP fluorescent marker. PCR primers were designed to discriminate between wild-type and knockout alleles, and individual colonies derived from single cells were genotyped (Fig. 5B). Sanger sequencing of PCR products of clones containing a wild-type allele was performed to exclude the presence of any indels or inversions. Multiple, independent disomic and trisomic clones of appropriate genotypes were isolated and subjected to *in vitro* B-cell differentiation. No significant difference in production of CD34^+^ cells was noted on comparison of disomic or trisomic clones, irrespective of *EDNRB* genotype (Fig. 5C). CD34^+^ cells were isolated from embryoid bodies and grown on MS5 cells for B-cell differentiation. After 21 days of MS5 cell co-culture, the number of CD19^+^ cells generated per input CD34^+^ cell was enumerated (Fig. 5D). Knock-out of *EDNRB* in trisomic cells had minimal effect on B cell production, as compared to trisomic wild-type or *EDNRB*^+/−^ clones. Disomic *EDNRB*^−/−^ cells generated roughly the same ratio of B cells as trisomic *EDNRB*^+/+^ or *EDNRB*^+/−^ cells, and more than trisomic *EDNRB*^−/−^ cells, although the difference was not statistically significant. *EDNRB* knockout in disomic cells reduced B cell production (p-value ≤ 0.15), with EDNRB^−/−^ cells generating fewer B cells than disomic *EDNRB*^+/−^ or *EDNRB*^+/+^ cells. Although, not statistically significant, coupled with shRNA results, this trend is consistent with the hypothesis that *EDNRB* serves a critical role in B cell differentiation.

## Discussion

T21 individuals exhibit reduced numbers of circulating B and T cells, which likely contributes to increased susceptibility to infections, although the mechanisms underlying these phenotypes are unknown^8–11^. Here, we differentiated human isogenic iPS cells into lymphoid lineages to gain insight into pathogenesis. We did not observe differences between disomic and trisomic cells in production of T cell progenitor cells. Given the reduction in T cells observed in T21 individuals^9^, we suggest that abnormalities in T cell generation *in vivo* may be extrinsic to hematopoietic progenitor cells. As thymi are smaller in size in T21 fetuses compared to age-matched controls^28^, we speculate that reduced T cell numbers *in vivo* may reflect a developmental patterning abnormality. If that were the case, *in vitro* T cell generation by trisomic iPS cells would be unaffected.

Our observations pertaining to reduced B cell production by trisomic iPS *in vitro* are reminiscent of decreased B cell development described in T21 individuals^8,10^. Transcriptome analysis of differentiated cells revealed that the Endothelin Receptor B (*EDNRB*), and its ligand (*EDN1*) were among the top 20 downregulated genes in trisomic clones (Supplementary Table 1). These observations were validated by qPCR, and subsequent bioinformatic analysis revealed overlap between endothelin signaling and G-protein receptor signaling gene sets and transcripts enriched in disomic clones. Endothelin signaling was initially recognized in relation to regulation of vasodilation, but has since been associated with numerous cell processes, including regulation of differentiation and proliferation depending on cellular context^24^. Limited data link endothelin signaling to regulation of hematopoiesis, or lymphopoiesis. However, it has been reported that *EDNRB* knockout mice have small spleens and exhibit impaired B cell development^25,26^. This phenotype correlates with our observations of reduced B cell development in trisomic clones exhibiting a reduction in *EDNRB* expression. Abnormalities in *EDNRB* expression has not previously been linked to T21. However, reduced *EDN1* expression has described in peripheral blood cells of T21 adolescents^29^. *EDNRB* is encoded on chromosome 13, while *EDN1* is localized to chromosome 6, and thus unanticipated candidates to mediate a trisomy 21 phenotype. Therefore, reduced expression is presumably the consequence of altered transcriptional networks in trisomic cells. Besides potential roles for transcriptional regulators expressed from chromosome 21, it is also possible that microRNAs encoded on chromosome 21 may ultimately influence *EDNRB* expression.

To establish a causal relationship between a reduction in *EDNRB* expression and defects in B cell development, we utilized two different approaches. Normal, disomic CB CD34^+^ cells were transduced with either specific shRNA targeting *EDNRB* or a scrambled shRNA control, and differentiated to generate B cells *in vitro*. In samples transduced with *EDNRB* shRNA, generation of CD45^+^CD34^−^CD19^+^CD10^+^ cells was markedly reduced, thus indicating that reduction of *EDNRB* expression is sufficient to impair B cell differentiation in a disomic cell context.

Further evidence of a critical role for *EDNRB* in B cell development was obtained through CRISPR/Cas9 targeting of *EDNRB* in disomic and trisomic isogenic iPS cells. No difference in percentage production of CD34^+^ cells was observed, irrespective of *EDNRB* genotype in either disomic or trisomic cells. The production of B cells quantitated as a ratio of number of B cells produced per input CD34^+^ cell indicated that disomic *EDNRB*^−/−^ cells generate fewer B cells/CD34^+^ cell than disomic *EDNRB*^+/−^ or *EDNRB*^+/+^ clones. Furthermore, disomic *EDNRB*^−/−^ clones were comparable in B cell production as trisomic *EDNRB*^+/+^ or *EDNRB*^−/−^ clones, indicating that *EDNRB* ablation confers a trisomic-like phenotype to disomic cells in terms of B-cell production. These findings strongly implicate reduced *EDNRB* expression in trisomic cells as a major contributor to reduced B cell potential, and provide a potential pathological basis for observations in T21 individuals. More broadly, our work illustrates how *in vitro* differentiation of human iPS can be employed to discriminate between intrinsic and extrinsic effects of altered genotypes on individual hematopoietic lineages.

## Experimental Procedures

### Maintenance and Differentiation of iPS Cells

Human isogenic iPS cells that are genetically indistinguishable with the exception of disomy or trisomy for chromosome 21 have been previously characterized^18^. iPS cells were maintained on irradiated CF-1 feeder cells (Global Stem) in DMEM/F12 media supplemented with 20% knockout serum replacement (KOSR), 1% L-glutamine, 1% non-essential amino acids, 0.1 mM β-mercaptoethanol and 10 ng/mL Fgf-basic (StemGent). *In vitro* hematopoietic differentiation via embryoid bodies (EBs) was performed following existing protocols^18,21^. In short, prior to differentiation, iPS cells were feeder-depleted by plating out for 24–48 hours on growth factor-reduced matrigel (Corning). Colonies were then treated with 10 mg/mL collagenase B (Roche) for 20 minutes at 37 C, followed by 0.05% trypsin for 1.5 minutes at 25 C. 500 μL of fetal calf serum (FCS) was added to stop trypsin digestion, and colonies were collected and washed with DMEM/F12 media supplemented with 5% KOSR. Resultant colony fragments were plated on ultra-low attachment plates (Corning) in base media of StemPro-34 media (ThermoFisher) supplemented with 1% L-glutamine, 5 mg/mL ascorbic acid, 150 μg/mL transferrin (Roche) and 4 × 10^−4^ M monothioglycerol. Cytokines were added on appropriate days (as shown in Fig. 1) at the following concentrations: BMP4 (10 ng/mL) (R and D Systems), FGF (5 ng/mL) (Stemgent), SB-431542 (6 μM) (Stemgent), VEGF (15 ng/mL), IL-6 (10 ng/mL), IL-11 (5 ng/mL), IGF-1 (25 ng/mL), SCF (50 ng/mL) (R and D Systems), EPO (2 U/mL), TPO (30 ng/mL), Flt-3L (10 ng/mL), IL-3 (30 ng/mL). Embryoid bodies were maintained in a hypoxic 5% CO_2_/5% O_2_/90% N_2_ environment for 8 days before transfer to an incubator with ambient oxygen for the balance of differentiation. After 9–10 days of differentiation, EBs were dissociated by incubation in 10 mg/mL collagenase IV in 20% FSC/PBS for 1 hour at 37 C, followed by 5 min incubation in 0.25% trypsin and passage through a 20 gauge needle. Single cells were stained with anti-CD34-APC (Biolegend) and CD34^+^ viable cells were isolated by fluorescence activated cell sorting (FACS) sorting. All cytokines were purchased from Peprotech unless otherwise noted.

### B-Cell Differentiation and Analysis of iPS Derived CD34^+^ Cells

MS5 cells were maintained in base media consisting of Mem-α (powder, Thermofisher) supplemented with 10% FCS (Hyclone) and 1% L-glutamine. 48 hours prior to co-culture, 40 000 MS5 cells were plated per well in 12-well dishes. After FACS isolation from EBs, CD34^+^ cells were added to MS5 cells in base media supplemented with monothioglycerol (4 × 10^−4^ M), SCF (50 ng/mL), G-CSF (25 ng/mL), Flt3-L (50 ng/mL) and IL-7 (20 ng/mL). Every 7 days, cells were given a half media change with fresh cytokines, and samples were collected after 21 or 28 days of co-culture. For analysis, co-culture supernatant was collected, and the MS5 layer was incubated with collagenase IV (1 mg/mL) for 20 minutes at 37 C followed by treatment with 0.25% trypsin for 5 minutes, after which this fraction was combined with the previous supernatant. For experiments, 4 disomic and 4 trisomic sublcones from the same parental line were differentiated in parallel. Multiple independent differentiation experiments were performed, and results were compiled for analysis (Fig. 1). P-values were calculated by Student’s T test.

### T-Cell Differentiation and Analysis

OP9-DL1 cells were maintained in base media consisting of Mem-α supplemented with 10% FCS and 1% L-glutamine. 48 hours prior to differentiation, 40 000 OP9-DL1 cells were plated per well in 12-well dishes. CD34^+^ cells were then added to OP9-DL1 cells in differentiation media consisting of Mem-α supplemented with 20% FCS, 1% L-Glutamine, Flt3-L (5 ng/mL), IL-7 (5 ng/mL) and SCF (50 ng/mL). Every 4–5 days, cells were collected, filtered through a 50 μm filter and plated on fresh OP9-DL1 cells^21^. For experiments, 4 disomic and 4 trisomic sublcones from the same parental line were differentiated in parallel.

### Lentivirus Production

For lentivirus production, HEK 293 T cells were grown to 80% confluence in DMEM supplemented with 10% FCS and 1% L-glutamine in 15 cm tissue culture plates. Cells were then transfected using 63 μL of 7.5 mM polyethylenimine, along with 3.5 μg of Δ8.9 packaging plasmid, 1.75 μg of Vsv-g envelope plasmid and 5.25 μg of appropriate vector. 60 hours after transfection, supernatant was collected, passed through a 0.45 μm filter, and concentrated by ultracentrifugation at 75 000 g for 2 hours at 4 C. After resuspension in DMEM media, virus was aliquoted and stored at −80. Virus was titered using a qPCR lentiviral titer kit as per manufacturer’s directions (ABM). shRNA constructs directed to *EDNRB* were purchased from Sigma-Aldrich, clone IDs NM_000115.2-1527s21c1 (shRNA1), NM_000115.2-1323s21c1 (shRNA2), NM_000115.1-1488s1c1, NM_000115.2-1659s21c1 and NM_000115.1-515s1c1.

### Differentiation and Transduction of Cord Blood CD34^+^ Cells

Cord blood (CB) CD34^+^ cells were differentiated to generate B cells as described above. For lentiviral transduction, cells were plated in a low adhesion 96 well plate with virus at a multiplicity of infection (MOI) of 25. 8 μg/mL of protamine sulfate was added, along with B cell differentiation media, to bring the volume of each sample to 100 μL. After 24 hour incubation at 37 C, samples were transferred to 12 well plates containing MS5 cells and 2 mL of differentiation media. Appropriate samples were supplemented with puromycin (1 mg/mL) for selection of transduced cells for the duration of differentiation. These experiments were performed 3 independent times in triplicate, and statistical significance was calculated by Student’s T test.

### Cell Staining and FACS

Cells were prepared and stained for FACS using standard protocols. Combinations of the following antibodies were used for all analysis. CD4-BV605, CD5-APC/Cy7, CD8-AF700, CD10-PE, CD10-PE-Cy7, CD45-PE-Cy7, CD34-APC, CD34-AF700, CD19-FITC, CD19-APC, CD3-FITC and dapi for viability. Antibodies were obtained from Biolegend. To obtain absolute counts of differentiated cells, CountBright beads (ThermoFisher) were added to stained cells. Cells were analyzed on a LSRFortessa (BD Biosciences), and sorted using a FACSAria II (BD Biosciences).

### CRISPR/Cas9 Editing

Single guide RNAs (sgRNAs) targeting *EDNRB* were selected using online tools (http://crispr.mit.edu). Several sgRNAs were tested for editing efficiency, and two guides (GTGCCAACGACCGCGCCAGAC, GTCTGGCGCGGTCGTTGGCAC) were selected for use in isogenic iPS cells. sgRNAs were cloned into px458 (Addgene) via golden gate cloning^30^. To perform editing in isogenic cells, 25 μg of vector containing each sgRNA was electroporated into 10 × 10^6^ undifferentiated iPS cells using a BioRad Gene Pulsar II at 250 mV with a resistance of 500 μF. After electroporation, cells were cultured on matrigel coated plates in mTESR media (Stem Cell Technologies) supplemented with 10 μM Y27632 (Stemgent) for 48 hours. Cells were then collected, and GFP^+^ cells were isolated by FACS. After sorting, cells were plated at limiting dilution to promote formation of colonies from single cells. Colonies were expanded for analysis before transfer to CF1 feeder cells for differentiation experiments. Editing was performed in disomic or trisomic isogenic cells, and 3 Ednrb−/− clones, 1 Ednrb+/− clone, and 2 Ednrb+/+ clones for each background were used for *in vitro* differentiation experiments. Multiple independent differentiation experiments were performed, and results were compiled for analysis (Fig. 5). P-values were calculated by Student’s T test.

### Microarray analysis

Disomic and trisomic isogenic iPS cells were differentiated in parallel, and after co-culture with MS5 cells, CD45^+^CD34^−^ CD19^+^ cells were isolated by FACS. RNA was extracted using Trizol. Global gene expression analysis by microarray was performed on the Human HG U133 Plus 2.0 platform. Affymetrix CEL files were normalized using RMA, followed by the *SVA* package for removing batch effects and other unwanted variation^31,32^. Differentially expressed genes were detected by *lmFit* function in *limma* package with a threshold of fold change ≥2 and adjusted p-value ≤ 0.05^33^. Gene Set Enrichment Analysis (GSEA) was performed using GSEA software (http://broadinstitute.org/gsea) with default parameters, and existing gene sets (PID_Endothelin_Pathway, G_Protein_Coupled_Receptor_Activity)^34^.

### Data availability

The datasets generated and analyzed during the current study are available in the GEO database, accession number GSE110064.

## Electronic supplementary material

Supplementary Information
