# Supplementary material for: Downregulation of Endothelin Receptor B Contributes to Defective B Cell Lymphopoiesis in Trisomy 21 Pluripotent Stem Cells
Source: Sci Rep. 2018 May 22;8:8001. doi: 10.1038/s41598-018-26123-y (PMC5964225; doi:10.1038/s41598-018-26123-y)
Supplement: Supplementary file 1 — Supplementary Information [file 41598_2018_26123_MOESM1_ESM.docx]

Downregulation of Endothelin Receptor B Contributes to Defective B Cell Lymphopoiesis in Trisomy 21 Pluripotent Cells

MacLean, Glenn A., McEldoon, Jennifer., Huang, Jialiang., Allred, Jeremy., Canver, Matthew C., Orkin, Stuart. H.

Supplementary Information


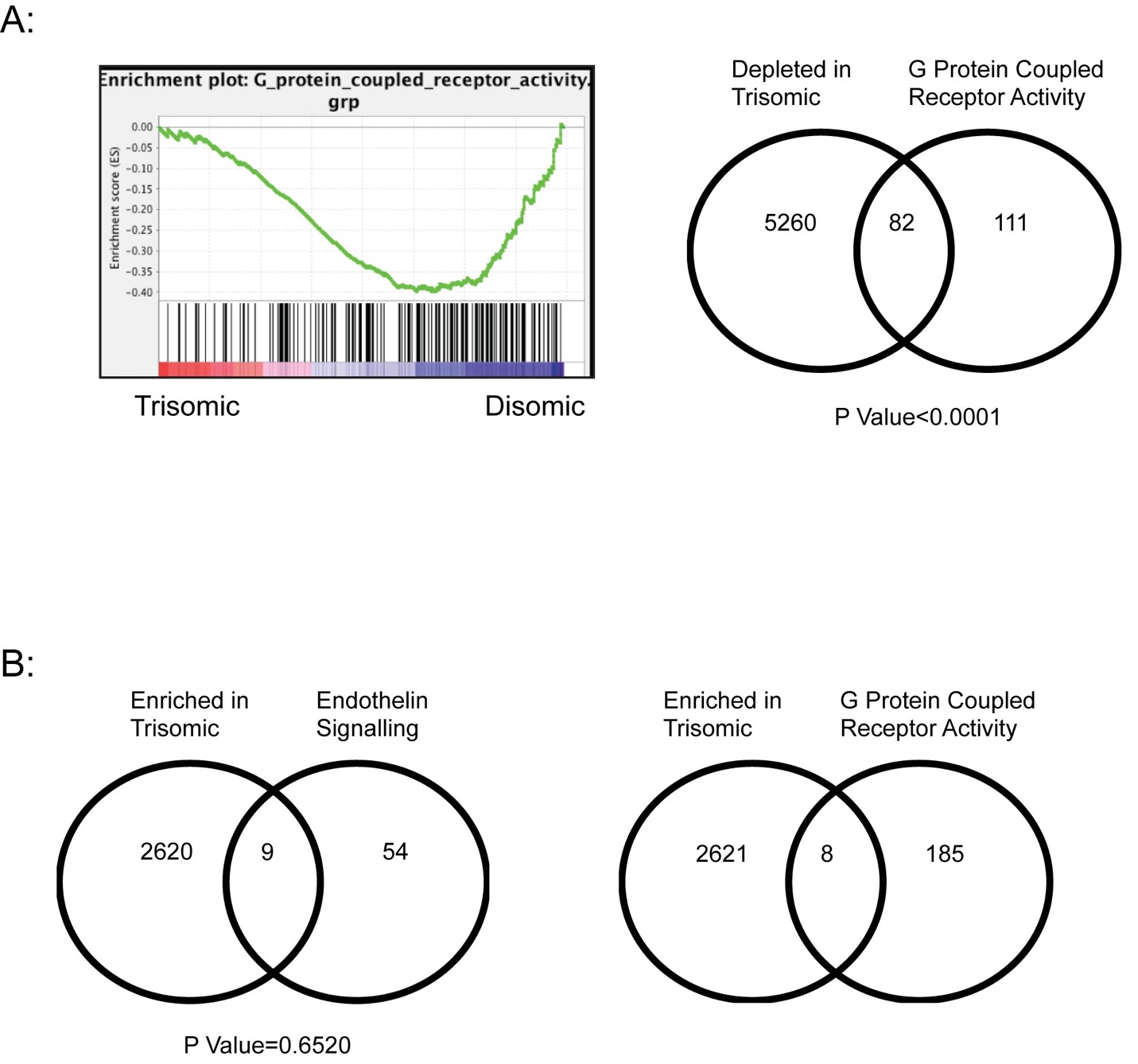


Supplementary Figure 1


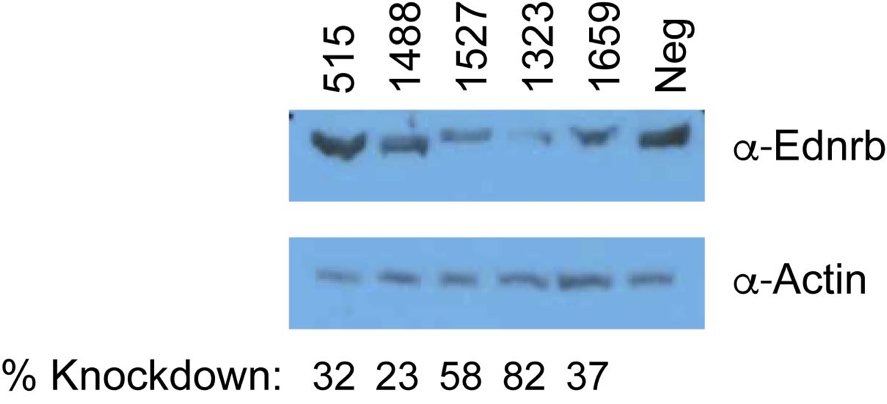


Supplementary Figure 2

Supplementary Figure Legends

Supplementary Figure 1

(A) GSEA of G_protein_coupled_receptor_activity gene set shows general

enrichment in disomic cells. A Venn diagram showing significant (p<0.0001,

Fisher’s exact test) overlap between genes depleted Log2(fold-change)<0.2 in

trisomic cells and G-protein receptor genes. (B) Venn diagrams showing lack of

significant overlap between genes enriched in trisomic cells (Log2(fold-change)>0.2), and Endothelin Signaling and G-Protein Coupled Receptor Activity gene sets.

Supplementary Figure 2

Western blot demonstrating knockdown of EDNRB in HEK293 cells after transduction with one of five shRNA targeted against *EDNRB*. Labels above the blot refer to shRNA ID numbers as described in the experimental procedures. After electrophoresis, and transfer to membrane, the blot was cut, and sections were hybridized with anti-actin (Santa Cruz), or anti-EDNRB (GeneTex) antibodies as appropriate. Knockdown efficiency relative to untransduced cells expressing endogenous *EDNRB* was calculated

using ImageJ analysis software.

Supplementary Table 1

Top genes depleted in differentiated trisomic cells compared to isogenic disomic

controls. Genes are ranked based on Log (Fold Depletion), and Adjusted P-Value is

shown. Endothelin receptor type B (Ednrb) and Endothelin 1 (Edn1) are highlighted,

and were selected for further study.

Supplementary Table 2

Top genes enriched in differentiated trisomic cells compared to isogenic disomic

controls. Genes are ranked based on Log (Fold Enrichment), and Adjusted P-Value is

shown.
